# The pandemic changes daily life and ways of living: technosociality and user/families experiences

**DOI:** 10.1590/0034-7167-2022-0177

**Published:** 2023-03-06

**Authors:** Leila Cristine do Nascimento, Tamires Carolina da Silva, Daniela Priscila Oliveira do Vale Tafner, Virgínia Junqueira Oliveira, Selma Maria da Fonseca Viegas

**Affiliations:** I Universidade Federal de São João del-Rei. Divinópolis, Minas Gerais, Brazil; II Universidade Regional de Blumenau. Blumenau, Santa Catarina, Brazil

**Keywords:** Pandemics, Technology, Social Networking, Primary Health Care, Life Change Events., Pandemias, Tecnología, Red Social, Atención Primaria de Salud, Acontecimientos que Cambian la Vida., Pandemias, Tecnologia, Rede Social, Atenção Primária à, Saúde, Acontecimentos que Mudam a Vida.

## Abstract

**Objectives:**

understand the changes imposed by the COVID-19 pandemic in the daily lives of users of Primary Health Care and their families and its impact on self-care and health promotion.

**Methods:**

this is a holistic-qualitative multiple case study, based on the Comprehensive Sociology of Everyday Life, in which 61 users participated.

**Results:**

experiencing a new daily life in COVID-19 pandemic times, users express their feelings, adaptation to new habits and ways of living. Health technologies and virtual social networks stand out in helping with everyday tasks, in communicating with loved ones and health professionals, and in validating dubious information. Faith and spirituality arise in the face of uncertainty and suffering.

**Final Considerations:**

it is imperative to pay close attention to the changes in daily life caused by the COVID-19 pandemic, in order to offer care directed to the singular and collective needs.

## RESUMO

**Objetivos:**

compreender as mudanças impostas pela pandemia de COVID-19 no quotidiano de usuários da Atenção Primária à Saúde e suas famílias e seu impacto no autocuidado e na promoção da saúde.

**Métodos:**

trata-se de estudo de casos múltiplos holístico-qualitativo, fundamentado na Sociologia Compreensiva do Quotidiano, no qual participaram 61 usuários.

**Resultados:**

vivenciando um novo quotidiano em tempos da pandemia de COVID-19, os usuários expressam seus sentimentos, a adaptação aos novos hábitos e modos de viver. As tecnologias em saúde e as redes sociais virtuais se destacam no auxílio às tarefas cotidianas, para a comunicação com entes queridos e os profissionais da saúde, e na validação de informações duvidosas. A fé e a espiritualidade surgem diante de incertezas e sofrimento.

**Considerações Finais:**

é imperativo um olhar atento às transformações no quotidiano causadas pela pandemia de COVID-19, a fim de ofertar um cuidado direcionado às necessidades singulares e do coletivo.

## RESUMEN

**Objetivos:**

comprender los cambios impuestos por la pandemia de la COVID-19 en el cotidiano de los usuarios de la Atención Primaria de Salud y sus familias y su impacto en el autocuidado y la promoción de la salud.

**Métodos:**

se trata de un estudio de caso múltiple holístico-cualitativo, basado en la Sociología Integral de la Vida Cotidiana, en el que participaron 61 usuarios.

**Resultados:**

experimentando una nueva cotidianidad en tiempos de la pandemia del COVID-19, los usuarios expresan sus sentimientos, adaptación a nuevos hábitos y formas de vivir. Las tecnologías de la salud y las redes sociales virtuales se destacan para ayudar en las tareas cotidianas, en la comunicación con los seres queridos y profesionales de la salud, y en la validación de información dudosa. La fe y la espiritualidad surgen ante la incertidumbre y el sufrimiento.

**Consideraciones Finales:**

es imperativo prestar mucha atención a los cambios en la vida cotidiana provocados por la pandemia de la COVID-19, a fin de ofrecer una atención dirigida a las necesidades singulares y colectivas.

## INTRODUCTION

The COVID-19 pandemic (COronaVIrus Disease-19) can be described as a major crisis that has qualified as one of the most important world-class health challenges of recent decades. It is an event that affects not only the biological, but the social and psychological disturbances of varying intensity for society^(1)^.

Due to the need to control the spread of the new coronavirus (SARS-CoV-2), the practice of social distancing was adopted. In Brazil, several measures were adopted that had an impact on changes in the daily lives of people and families. A considerable part of workers began to develop their activities at home. There was a reduction in physical activity, leading to an increase in sedentary behavior time. People spent more hours on TV and the internet. Constant too were changes in eating habits^(2)^.

Public health crises, such as the COVID-19 pandemic, bring with them great stress, concern and anxiety for society. The sudden change in the daily lives of people and families came loaded with fears and anxieties, against the backdrop of uncertainty and unpredictability. Indeed, this pandemic poses new challenges: the identification of invisible damage; the damage caused to mental health; depression; high levels of stress and anxiety^(1)^.

It appears that the role of technologies and virtual social networks amidst the pandemic was significant, due to the need for isolation/social distancing. Thus, it is important to take a look at the changes in people’s and families’ daily lives, given the experiences in COVID-19 pandemic times and use of technosociality, which can be delineated as users’ socialization through technologies, particularly those related to communication^(3)^. Sociologist Maffesoli characterizes everyday life as the way of living of individuals and the collective^(4)^, constituting the cause and effect of social interactions^(5)^. Through the context of the pandemic, the internet promotes the sharing of tastes, religious or cultural, albeit virtually. In this sense, we can say that the confinement lived consolidates the postmodern tribes, once this health crisis has been mitigated or resolved, these tribes will surely develop^(6)^.

Understanding changes in everyday life and ways of living in COVID-19 pandemic times is especially relevant in the face of a situation of incomparable magnitude. Having said this, the question is: what are the feelings and experiences of Family Health Strategy (FHS) users during the COVID-19 pandemic? How does technosociality show itself in FHS users’ daily lives in COVID-19 pandemic times? What strategies adopted by FHS users to cope with the situations imposed by the pandemic?

## OBJECTIVES

To understand the changes imposed by the COVID-19 pandemic in FHS users’ and their families’ lives and their impact on self-care and health promotion.

## METHODS

### Ethical aspects

This study was approved by the Ethics Committee of the *Universidade Federal de São João del-Rei*, Midwest Campus. National Health Council Resolution 466, of December 12, 2012, and Resolution 510, of April 7, 2016, which regulate the guidelines and standards to be followed by research involving human beings, were respected^(7-8)^.

### Theoretical-methodological framework

The holistic multiple case study^(9)^ was used as a methodological framework and Michel Maffesoli’s Comprehensive Sociology of Everyday Life^(10)^ as a theoretical framework.

The holistic multiple case study provides an intense analysis of the object of investigation, grouping numerous information rich in detail, aiming to understand the reality to be studied. It is noteworthy that studies with multiple cases rely on the logic of replication, so, after revealing significant findings in the first case, they are replicated in other cases, one of the reasons for being considered more robust and convincing^(9)^.

In turn, Michel Maffesoli’s Comprehensive Sociology of Everyday Life indicates understanding rather than explaining social phenomena, valuing everyday knowledge, proposing an open and sensitive reason in the face of lived experiences and feelings^(10-11)^.

### Study design

This is a qualitative study. This research met the COnsolidated criteria for REporting Qualitative research (COREQ) recommendations.

The qualitative approach aims to understand the phenomena in everyday life, considering each individual’ uniqueness; however, understanding that their experience takes place in the collective context; therefore, the culture of the groups to which they belong reflects and contextualizes their experiences^(12)^. The qualitative approach in this study becomes opportune, because it is the investigation of individual experiences, emotions and feelings as well as manifest behaviors^(13)^.

### Study setting

This study contains multiple cases, defined by the scenarios of two cities in the Extended Health Region of West Minas Gerais and one city in the Health Region of the Middle Valley of Itajaí, in the state of SC, Brazil. Participants of this research are SUS users registered and followed up by FHS teams and who were aged ≥ 18 years and who could answer for themselves. Some condition that makes it impossible for people to verbalize if they made an exclusion criterion.

Users of 1^st^ and 2^nd^ cases were contacted remotely, due to the need to maintain social distancing as a preventive measure for COVID-19. Initially, FHS team professionals and professionals indicated as key informants of a Reference Center for Elder Health in a city of SC were asked to provide users’ contacts, sent after users’ approach and prior consent. Some contacts were acquired by indication of another participant, but most indications were from professionals. The invitation was made using electronic means, such as by email or WhatsApp, with up to five attempts to communicate/contact with users.

Therefore, for the remote capture of possible participants in the 1^st^ and 2^nd^ cases, the snowball methodological technique was used, which comprises a non-probabilistic sampling technique, in which participants’ indications are derived from other participants, i.e., the first interviewees indicate others, and so on, until data saturation is reached^(14)^.

In the 3^rd^ case, with the flexibility of social distancing measures, the research was conducted in the FHS units, with the selection being made randomly by means of a lottery, totaling six FHS units and a total of eight teams. Participant gathering took place in the units’ waiting room, and the interview was held in a private room after presentation of an Informed Consent Form and recorded in audio. It is emphasized that the recommended measures to control the COVID-19 pandemic were respected.

### Data source

Intensive open-ended interview and field notes were subdivided into theoretical, interaction, methodological and reflexive notes^(15)^. The open-ended interview, with a semi-structured script, contained questions aiming at characterizing participants, in addition to 13 open questions aiming to understand the object of study. In turn, the above-mentioned notes were used to detail characteristics of the research setting, relevant facts of data collection and for the operational procedures of the research.

### Data collection and organization

Data collection took place between April and October 2021. A total of 61 people participated in this study, 54 female and seven male. A total of 116 users received the invitation to participate, but 55 users refused to participate. The interview lasted approximately 19 minutes. Participants’ mean age was 47 years, 27 individuals declare to be married, representing the predominant marital status, 47 participants have their own home. Regarding education, complete high school was predominant, declared by 19 people, the 3^rd^ case had more impact on this variable. Of the total number of participants, 38 reported having a fixed monthly income, the mean income of these informants being R$1,488.77 (about US$270.68), and the highest income was found in the municipality of 1^st^ case. The mean daily time of use of social networks and internet technologies was approximately 4.67 hours. To ensure participant anonymity and information confidentiality, the letter “I” (interviewee) was used, sequenced in the numerical order of the interview.

### Data analysis

**Figure 1:**
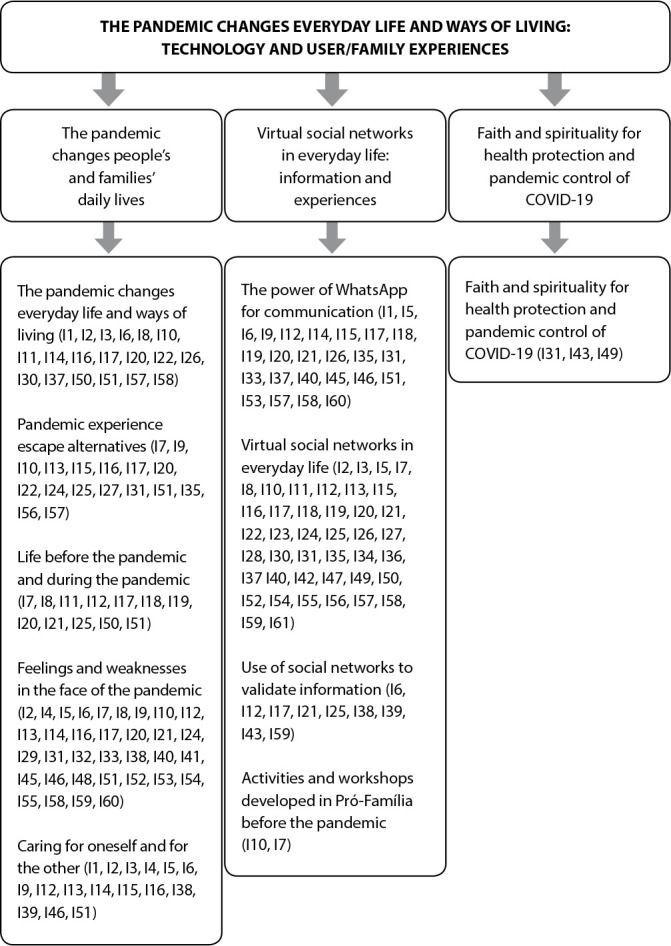
Thematic category, subcategories and Registration Units with saturation by literal replication of cases 1, 2 and 3, 2021

For analysis, the analytical technique of cross-synthesis of cases^(9)^ was followed, adopting thematic content analysis, established by semantic criterion^(16)^. The unit of analysis was “technosociality and health promotion in FHS users’ daily lives in COVID-19 pandemic times”. The analysis originated three thematic categories presented in Figure 1. This article covers the category The pandemic changes everyday life and ways of living: technosociality and experiences of users and families.

## RESULTS

Figure 1 describes the thematic category, the three subcategories and the Record Units with literal replication saturation.

### The pandemic changes people’s and families’ daily lives

The pandemic is presented as transforming the daily lives of people and families, emerging in everyday life feelings of isolation/social distance and absence of being-together. New habits and changes in ways of living are emerging:

*Today, amidst the pandemic, you can solve a lot of things at home, some payment, if you have something in your health, you can solve it by calling or by WhatsApp or by email* [...] *because of the agglomeration, this virus it exists. I’m not so afraid of this virus, thank God, I didn’t catch it, but just like that* [...] *it exists! So, we have to be careful, we have to prevent it, and how are we going to prevent it? Taking care of yourself at home, but we have our chores, we have day to day things, so you can solve this not by going there, but using technology.* (I2)

*I had to stop working, because my daughters wanted me to stop working because of COVID, and I did, but now I’m going for an interview, I’m going back to work, I’m going to get busy again. To go back* [...] [pause] *I don’t know* [...] *to be people!* (I6)

*In this pandemic, everyone living in the same house together, isn’t it? We learned to awaken this love, to give more value to our family, that we sometimes give more value to outsiders than to those inside, and this made us reflect a lot. This pandemic made us see how much we need each other, didn’t it? Because being alone is the worst thing ever.* (I57)

*So, it’s been a very difficult time for me, so, before alone, I’m still alone, but, well, people’s heads are racing! So, this face with the internet is being very cool, because I get a lot of calls, yesterday was a day when there were only people calling. How long am I on the internet? Look, if there’s something interesting, I stay almost a whole day, I participate in the programs I like.* (I10)

Changes in everyday life are accompanied by the need to adapt and develop new skills:

*This first contact was very difficult, this access to this freedom that I’m having now talking to you, it was difficult, because it’s a totally innovative thing, isn’t it? It is a new situation for older adults, especially those who were not born pushing buttons. Our generation was the generation that was everything! It was the first television, it was the first color television, it was the telephone. Our generation has entered this era of everyone, of everything. So, it wasn’t that simple, now it’s been a little.* [...] *look, with the pandemic, social media came to favor, but in-person classes are definitely better, because there is eye to eye, there is! There is contact, there is a hug, there is much more than what you have now, but this moment requires this change, it is also well accepted, you know? We are doing our best to live within normality, to lead a more or less normal life.* (I8)

*Nowadays, you need physiotherapy, you can’t find a physiotherapist because of COVID,* [...] *this is absurd, because the person has not stopped depending on the SUS, 98% of Brazilians today depend on the SUS, so, if it stops everything, who will help the other things? So, I think social networks are an incentive. Ah, but there is no way for the physical therapist to go home. Oh, really? But there’s a way for him to give me online class for person. He doesn’t really need to be there, he has to have a relative, he really teaches the correct exercises for the person, for the person to do on the other. I think it’s effective, understand?* (I16)

The pandemic has implications for users’ dietary patterns:

*Because, in this pandemic, it only made a lot of people fat, didn’t it? Eat this, do this. “Oh, I’ll look for this on the internet”, but everything is fat, nothing light!* (I11)

In the new routine, there were changes in the work or in the way of carrying out the work, remotely with overload:

*I confess that I am very tired, because I end up having to spend a lot of time* [on the internet] *and this has been intense for some years and, with the advent of the pandemic, this has increased, increased* [...] *especially online meetings* [...] *here come these lives, these tireless meetings, for me, who already spend all my time on the internet, on Facebook, Instagram and WhatsApp, which are these three that consume me, plus the online meetings, this has been tiring me a lot. Honestly, if I could, if it wasn’t for this new reality, I would hang up the phone* [laughs]. [...] *I doubled my work at home, doubled the number of online meetings, doubled lives, doubled everything online, doubled my time, doubled my time on social media and the internet, doubled my time in front of the screen, tired my eyes, tired my mind, tired everything. (I51)*

Several changes occurred in the daily lives of participants of this study in COVID-19 pandemic times: isolation/social distancing, according to them, brought loneliness, especially for older adults; there was a need to adapt to technology to assist in everyday activities; new eating patterns were noticed. In the population of young people and young adults, reports predominate about the increase in work overload, enhanced by the constant use of technology; however, technology was referenced to facilitate access to health (FN).

Users used alternatives to escape from everyday life in this pandemic, due to the need to maintain good mental health:

Look, I try in my free time to be with my family, I try even if it’s from a distance in a meeting, just like we’re doing through Google Meet, I try to do activities, even if they distract me, relax me, like, for example, listening to music, cooking, which I like. So, I try to do activities that distract me a little from what is being experienced because of the pandemic. I like to read books too. (I17)

*I tried to get back to physical exercise, so I could have an extra distraction, because I take all the care with my mask, distancing, I always really liked exercising, but at the beginning of the pandemic, in that first year, we were practically not leaving the house, then it was really lockdown and we were really stuck inside the house, so I looked for what was possible, get back to physical exercise, leave social media a little, try to read a book, study a little more, enjoy this free time that I ended up staying, right? With free time, because I am unemployed, so I tried to reverse this in the best way possible, try to make the most of this time, taking care of my health, my mind and trying not to leave a gap* [...] *as I already had depression, not allowing for a new crisis, being attentive too* [...] *I was very attentive to all the signs when I was depressed.* (I24)

Users carry out activities to help them relax or escape the pandemic’s stressful climate as well as use of social networks for fun and communication with friends and family (FN).

Life before and during the pandemic points out the nuances of a new daily life. when experiencing the challenge of isolation/social distancing and the changes in interacting with others, family members and health professionals:

*It’s not good, I think it’s harmful, but then there are days when I don’t talk to anyone, you know? So sometimes I even find myself talking to myself.* [...] *we are in three sisters, my sister lives in another neighborhood and she usually calls me every day at night or I call her, it’s hard for me to go a day without calling, so it’s the only thing I have contact with someone, let’s say, with someone else, otherwise I spend the whole day alone. So, there on social networks, as they say, even not talking to anyone, like games, YouTube, something I watch to have an interaction.* (I12)

*I think the pandemic came a lot to show that what we had before was not working, for example, face-to-face groups. They were very focused on diabetes, hypertension and smokers, and now they have stopped, but I was never able to participate, because it was one o’clock, two o’clock in the afternoon, it would be unfeasible, so I think technology can help at that point.* (I17)

*When we need something related to health, whether it’s consultation, information, medication, anything in that sense, a lot has been done by this telephone intervention, we had this habit of going to the place, not now, before knowing about such a thing, before going, call!* (I20)

Through the pandemic, feelings and weaknesses emerge in the face of isolation, loneliness, longing and uncertainties:

*My goodness! This is something that is not easy to talk about, is it? Because* [...] *a lot of sadness, a lot of fear! Look* [...] [pause] *speechless* [...] [crying] *a lot of feeling* [...] *a lot of fear* [...] *I miss my grandchildren a lot* [...] *I miss my friends a lot* [...] *the things we used to do* [...] *so, that was really sad!* [cry]. (I6)

*I started to love the internet when I had COVID.* [...] *so, it was a phase when suddenly no one else even rang the bell here at home, because they were going to pick it up. I panicked, real panic, I was very afraid and if it wasn’t for the news I saw and if it wasn’t for my stepdaughter, if it wasn’t for my children, if it wasn’t for the moments of leisure, good news, alive, I don’t know if I could handle it, I, in particular, think it’s a health promotion, because, before, where I used to go, where all the old people go* [Pró-Família] [...]. *So, if it weren’t for all that, both me and my friends, I don’t think we would be able to handle it.* (I10)

*Wow, it affected me a lot* [...] *it started to affect me, that’s when I got into social networks and my self-esteem went downhill, and I started having a very strong anxiety crisis, I had to start therapy, thank God, therapy helped me a lot* [...]*, but it affected me very negatively, especially in the first year of the pandemic, when we didn’t see any solutions, right? Seeing multiple deaths, only increasing the number.* (I24)

Caring for oneself and for the other is pointed out as a constant concern. Due to the condition of social distancing, social networks and health technologies emerge as tools to assist in this care:

*Look, I try to talk as little as possible, I try to send positive messages and, in that sense, I think I’m taking care of myself and taking care of the other too.* (I5)

*I stay indoors more, you know? I hardly go out, when I go out on the street, I put on a mask. I’m not visiting my family. My family isn’t visiting me, you know? To, as they say, prevent, us and our family, neighbors too, I don’t go to the neighbors’ houses, so we are more homemade than anything, even the expense has increased* [laughs]. (I15)

### Virtual social networks in everyday life: information and experiences

The power of WhatsApp for establishing communication, especially between the family and the health team:

*primordial! I live close to the health center, but sometimes I even send a message on WhatsApp in private, they answer me, I don’t even have to call or go there.* (I21)

*Contact with family members, with the pandemic, we stay away. It’s through social networks that we have more contact nowadays, it’s the only way we can feel good and feel connected with the people we love.* (I26)

Thus, using virtual social networks in everyday life becomes for different situations, including information validity:

*In the beginning, last year, when there was this isolation thing, where no one goes out, it was a little complicated, because we have the mothers’ club, which are ladies who get together once a week with a nice coffee, with chat, with lots of laughs, all of this was sorely missed.* [...]*, but over time we were able to reconcile this chat online as well. Is not the same thing! they will never be!* (I8)

*I use the internet for everything. Do I have any doubts? Internet, so I use it, I look for home remedies, recipes* [...] *friendship, even to comment on where we went, if it was nice, express some feeling.* (I40)

*I try to see everything when I have any doubts, a friend says, “Oh, why don’t you take this?”, then I go there on the internet, I go there on Google, anyway, on YouTube. I like to be updated.* (I6)

*I always seek to look for the basis of information. The information that comes to me through a relative, without the scientific note of what was said, without the official source of that particular news, I try to search, when it is not possible to see this source, I search through other sources, such as Facebook, YouTube, through WhatsApp with other people who may be related to this type of news.* (I25)

*Fake news! Fake news, that one* [...] *we can’t just accept it at first. Especially when it’s something you’re going to ingest, or you’re going to apply to yourself.* (I39)

In case 1, workshops are developed by *Fundação Pró-Família* (Pro-Family Foundation):

*It is the Pró-Família that I attended all this time, where there are doctors who took care of me, where we did our craft work, everything you can imagine from handicrafts, paintings, ceramics, all of this at Pró-Família, which is a continuation of the health center. (I10)*

*I have a lot on WhatsApp, from Pró-Família too, I take care of myself because of that, which has the health part, the memory she sends* [pedagogue]*. I’ve also had an online class via WhatsApp on natural remedies, how to make tea, herbs* [...] *I think it helps a lot. (I7)*

*You have contact with the geriatrician, which is online, you have contact with the Yoga and Meditation teacher and physical activities that are online, since March last year* [2020]*, and I talk to 90% of people on WhatsApp daily* [...] *the physical activities on Pró-Família are live and are also recorded, then you choose the time and moment to do it, you have that freedom.* (I8)

*Fundação Pró-Família* was mentioned by older participants. This foundation has programs aimed at children and adolescents, older adults and organized voluntary groups, with 44 autonomous groups distributed in the neighborhoods of the municipality of case 1. It serves around 5,900 older adults through the *Programa Pró-Idoso* (Pro-Older Adult Program), of which 1,700 are monitored at the institution itself, and 4,300 in the 78 external centers. Older adults have access to leisure, sports, culture and citizenship activities, offered in 44 workshops. Additionally, a special program of tours, games and activities is offered throughout the year. In the most critical phases of the COVID-19 pandemic, activities were being carried out through virtual social networks (FN).

### Faith and spirituality for health protection and control of the COVID-19 pandemic

Faith and spirituality emerge in the reports, linked to health protection and control of the COVID-19 pandemic:

*I have a habit of praying and handing everything over to God, making sure He controls everything. Despite the pandemic, we think that evil does not come on God’s side, but God allows evil. So, I learned to rest and trust in God and whatever happens is His will.* (I31)

*I pray a lot, I ask God a lot for protection for me, my family, my children, my grandchildren, I always talk to them a lot to be careful.* (I43)

*To tell you the truth, I wasn’t afraid to take it, because I always trusted God, I always put him first in my life, I wasn’t afraid to tell the truth. I always wore a mask, washed my hands.* (I49)

Spirituality was present in the speeches of many research participants. It was noted that spiritual resources are used as an aid to overcome moments of fear and anxiety and to bring meaning to suffering (FN).

## DISCUSSION

Faced with the COVID-19 pandemic, the daily lives of people and families are changing, and new living standards and habits were necessary to adapt to preventive measures. Social media have played an essential role in providing information; people have increased their use of social media in order to keep in touch with people close to them and seek out more health information^(17)^.

A survey carried out in Brazil, with 45,161 individuals, aimed to describe changes in lifestyles in the most restrictive period for control of the COVID-19 pandemic. It pointed out that, among smokers, who represented 12% of the population studied, 34% increased their consumption of tobacco during the pandemic and 17.6% reported greater consumption of alcoholic beverages during social restriction. On the other hand, consumption of healthy foods decreased, while consumption of unhealthy foods increased. Physical activity in the pre-pandemic period was 30.1% among adults, however, during the pandemic, the rate became 12.0%. In relation to the mean time of TV use, there was an increase of 1 hour and 45 minutes compared to before the pandemic, totaling 3.31 hours. In turn, computer or tablet use increased by 1 hour and 30 minutes, adding up to more than 5 hours during the pandemic. The findings portray the increase in health risk behaviors^(2)^.

Another study, with 1,210 participants from 194 Chinese cities, assessed the psychological response of the population during the COVID-19 pandemic. It was observed that, of the total number of respondents, 24.5% reported minimal psychological impact, 21.7% assessed the mild psychological impact and 53.8% reported moderate or severe psychological impact. It was significantly associated with poor or very poor self-rated health status, with a greater psychological impact of the outbreak. Of those surveyed, 93.5% used the internet as a source of health information about COVID-19 and 75.1% were satisfied with the amount of health information available^(18)^.

As pointed out in a study developed in China, the dissemination of reliable information regarding the pandemic can have a positive impact on individuals’ practices, attitudes and knowledge about COVID-19^(19)^. Therefore, in a context in which information can impact caring for oneself and for the other, the quality of what is transmitted becomes particularly important.

A study carried out in Wuhan, after the health crisis installed by COVID-19, pointed out that, of the individuals surveyed, 19.2% had moderate to severe symptoms of depression. The pandemic represents a stressful time for the world population, with a significant increase in use of social networks and changes in health-related behavior. Individuals around the world have used social media more to obtain information about the COVID-19 pandemic. Individuals who have higher levels of anxiety tend to use virtual social media excessively. Social media provides users with choices for access and suitable place to visit, which image to create and decide who to interact with as well as momentarily escaping negative feelings^(17)^.

Although internet and technology use has become more intense with the pandemic, one of the changes perceived in daily life, especially among older adults, was the need to adapt to this use, becoming a means of replacing activities that were previously carried out in person, technologies that were not so present in older adults’ daily lives. Although, although technologies are increasingly widespread, their use still occurs mostly by young people, as older adults’ involvement is still restricted^(20)^. The recommended measure of social distancing has made technologies indispensable in people’s and families’ daily lives, imposing adaptations and changes in everyday life.

Thus, the interaction between people, in the pandemic context, was favored by using social networks and community sites, but it gave rise to a paradox of contamination thanks to the speed of cybernetic culture, which the effects can already be measured. An imaginary in the (re)creation of technological development allowing living together without old myths in a virtual environment^(3)^.

The results of this study pointed to changes in eating habits and physical activity and increased internet use. These findings corroborate a survey carried out in the United States, which found an increase in the habit of using the internet among adults during the pandemic. It is believed that the increase in screen time is favoring a greater caloric intake and decreasing physical activity, precisely because of the easier access to food at home and the ease of fast food, favoring substantial weight gain^(21)^. Weight gain may also be associated with increased anxiety and depression, which contribute to emotional hunger and compulsive overeating^(22)^.

The care of the self and the other appears in the voice of users of this study as precautionary measures against the possibility of contamination. The main precautionary measures taken by 1,210 individuals in a study conducted in China, are emphasized in 66.6% of hand washing after touching possibly contaminated objects, with 56.5% cleaning with soap, 59.8% using a mask frequently, 57.4% covering their mouths when coughing or sneezing, 41% washed their hands after coughing and 41% avoided sharing utensils. About 84.7% of respondents stayed at home for 20 to 24 hours a day in order to avoid COVID-19^(18)^.

In relation to older adults, it is observed that the effects of social distancing and loneliness, as well as the fear of death, loss of family members and hopelessness, become even more expressive factors, given the observation of a gradual trend towards greater family segregation with the pandemic, producing negative feelings and emotions and strengthening the feeling of loneliness. Furthermore, with older adults being the age group most vulnerable to disease severity, an increase in anxiety in the pandemic is expected. It should also be noted that the challenge of older adults complying with social isolation/distancing can be either aggravated or attenuated by psychological factors, among them: “aspects linked to typical developmental changes in cognitive and behavioral terms, in addition to cognitive inflexibility that increases with aging”^(22)^. In this scenario, technologies allow safe interaction, easing the feeling of loneliness and the complete interruption of social interactions. However, older individuals have limited access to certain internet services and more technological and complex devices, so only a fraction of older adults benefit from such service^(22)^.

In a context of crisis with the COVID-19 pandemic, spirituality emerges, in the voice of users of this study, as a power to face such a challenging situation. It should be noted that “spirituality is capable of mobilizing energies and positive attitudes that have unlimited potential in promoting people’s quality of life”^(23)^. Spirituality can be configured as a resource for coping with overwhelming circumstances and with a strong emotional impact^(24)^. Bringing meaning through spirituality helps to tolerate debilitating feelings and emotions, such as stress and anxiety^(23)^. In addition, in the face of numerous changes in the daily lives of people and families, the pandemic has proved, for some people, as an opportunity for religious deepening^(25)^. “Perhaps this is what makes the tragic environment experienced in everyday life, rather than slow, aware that there is a resurrection in progress. Resurrection in which it is in being-together, in being-with that the spiritual invisible will occupy a prominent place”^(26)^.

Due to the changes presented in the daily lives of people and families in the pandemic, established knowledge and published opinion demonstrates that in addition to the superficiality of things, there is something deeper that ensures, (re)affirms living together on solid foundations and legitimizes an understanding of the experience of the moment in its entirety, which is fundamental^(3)^.

People’s and families’ daily lives in COVID-19 pandemic times was revealed in feelings and emotions, in faith and spirituality. In everyday life, nothing can be considered banal and unimportant, small attitudes, feelings and notions reveal what has been lived, which can be understood by an attentive and sensitive eye.

### Study limitations

Although the intentional sample is a limitation in this study, it can be considered representative in populations with similar situations, due to the methodological design used when replicating the multiple cases.

### Contributions to nursing

The study brings contributions to nursing and the health area, by understanding that the pandemic changes the daily lives of users and families accompanied by FHS teams and reference professionals, impacting health through new habits and ways of living, providing health risk behaviors.

## FINAL CONSIDERATIONS

COVID-19 imposes changes in people’s and families’ daily lives, emphasizing technosociality. Technology is present to assist in everyday activities, from the most basic to the most elaborate, from recreation to remote work and health monitoring. Particularly, older adults, experiencing social distancing and fear of the disease, feel alone, expressing anxiety.

It is worth mentioning that, although the technology provides several benefits and the possibility of continuing activities performed previously, its excessive use can be a factor in illness. Excess information, especially untruths and increased work overload, significantly impacted the mental health of participants in this study. Moreover, users pointed out alternatives to escape from the pandemic in order to maintain mental health. Spirituality and faith emerge in the interviews as a support to better live in the face of tragic events due to morbidity and mortality by COVID-19. Impactful changes in daily life were reflected in health risk behaviors, such as inadequate eating habits and a sedentary lifestyle.

Technosociality stands out as a possible means of promoting the health of users and families, as opposed to favoring illness, especially related to excessive and inappropriate use. It is imperative to pay close attention to the transformations in everyday life, caused by the COVID-19 pandemic, in order to offer care directed to the singular and collective needs.
